# Use of Anti-*Aedes aegypti* Salivary Extract Antibody Concentration to Correlate Risk of Vector Exposure and Dengue Transmission Risk in Colombia

**DOI:** 10.1371/journal.pone.0081211

**Published:** 2013-12-02

**Authors:** Berlin Londono-Renteria, Jenny C. Cardenas, Lucio D. Cardenas, Rebecca C. Christofferson, Daniel M. Chisenhall, Dawn M. Wesson, Michael K. McCracken, Daisy Carvajal, Christopher N. Mores

**Affiliations:** 1 Louisiana State University, Baton Rouge, Louisiana, United States of America; 2 Hospital Municipal de Los Patios, Los Patios- Norte de Santander, Colombia; 3 Universidad de Pamplona, Pamplona, Colombia; 4 Tulane University, New Orleans, Louisiana, United States of America; Metabiota, United States of America

## Abstract

Norte de Santander is a region in Colombia with a high incidence of dengue virus (DENV). In this study, we examined the serum concentration of anti-*Aedes* salivary gland extract (SGE) antibodies as a biomarker of DENV infection and transmission, and assessed the duration of anti-SGE antibody concentration after exposure to the vector ceased. We also determined whether SGE antibody concentration could differentiate between positive and negative DENV infected individuals and whether there are differences in exposure for each DENV serotype. We observed a significant decrease in the concentration of IgG antibodies at least 40 days after returning to an “*Ae. aegypti*-free” area. In addition, we found significantly higher anti-SGE IgG concentrations in DENV positive patients with some difference in exposure to mosquito bites among DENV serotypes. We conclude that the concentration of IgG antibodies against SGE is an accurate indicator of risk of dengue virus transmission and disease presence.

## Introduction

In order to be transmitted, arboviruses need to infect the arthropod salivary glands and be secreted into the saliva. During the process of feeding, saliva mixed with viral particles is deposited at the bite site [1], [2]. Previous evidence has shown that the presence of saliva at the virus inoculation site may enhance or impair the establishment of infection [3], suggesting that the salivary proteins themselves play a role in the transmission of vector borne diseases. It has been shown that people living in malaria endemic areas presented higher IgG and IgM antibody concentration against the salivary proteins of the major vectors than people living in non-endemic regions, showing a positive correlation between antibody reactivity to vector saliva and disease transmission [4], [5]. Additionally, subjects with malaria or with clinical leishmaniasis presented higher IgG antibody concentration to vector saliva than healthy subjects living in the same region [6], [7].

Colombia is endemic for all four dengue virus (DENV) serotypes (DENV1, DENV2, DENV3, DENV4), with more than 90,000 cases of dengue cases reported in the territory by June of 2010, approximately 7,000 of which progressed to severe disease [8]. Norte de Santander is one of the regions with a high DENV index within Colombia [9]. In this region, more than 4,000 total cases of DENV were reported, 700 of which were severe cases [9]. Since no vaccine is currently available for DENV, control of the DENV vector, *Aedes aegypti (Ae. aegypti)*, remains one of the main mitigation strategies [10]. Traditional entomological methods to estimate vector-human contact rely mainly on efficient collection of vectors in the field, [11], [12] but this technique is highly biased by either mosquito collection methods (adults and aquatic stages), budget constraints, or other anthropological factors [13]. Thus, there is a need for epidemiological tools to better estimate vector contact rates and to subsequently correlate this to the risk of pathogen exposure and disease transmission.

Previous studies have shown the usefulness of antibodies against *Aedes sp*. salivary proteins as a marker for vector bite exposure and disease severity [14]–[16] but an evaluation of vector bites exposure for each DENV serotype has yet to be published. In this study we do not only evaluated the usefulness of anti-*Ae. aegypti* salivary gland extract (SGE) antibodies as a marker of risk for DENV infection and transmission in Colombia, but also the correlation of anti vector-saliva antibody concentration with regards to the infection with individual DENV serotypes. Additionally, we tested whether the duration of the anti-SGE antibody concentration in subjects exposed to mosquito bites change as they move between areas with and without *Ae. aegypti*.

## Materials and Methods

### Ethics Statement

Written permission for this study was requested and granted by the Local Institute of Health of Norte de Santander (Instituto Departamental de Salud). The protocols and research methods for these studies were reviewed and approved by Universidad de Pamplona, Los Patios Hospital and the Ethics Review Board of Hospital Erasmo Meoz specifically. The inclusion of febrile individuals was reviewed for the involved hospitals. In addition, the inclusion of healthy individuals was reviewed and approved by the University of Pamplona ethic board. The investigation was clearly explained to each individual and a written informed consent was obtained from each participant from Pamplona before collecting samples. The Local Hospital of Los Patios donated the serum remnants after test were performed in the facility with no identifiable information from the subjects (only age and gender was provided). Serum samples were collected in compliance with regulations on human subjects from both Colombia and United States.

### Study Area

The State of Norte de Santander is the principal area of commerce with Venezuela and the Caribbean; consequently agriculture is one of the main sources of income. The city of Pamplona is located in the northeast of the country at 2,342 meters above sea level (m.a.s.l.) (Average temperature: 16°C (high 24°C and low of 9°C). According to the National Statistic Administration Program (Departamento Administrativo Nacional de Estadistica), Pamplona has a population of approximately 105,780 in an area of 1,176 km^2^. There is not reported presence of *Ae. aegypti* in Colombia above of 2,200 m.a.s.l. [17]. Plamplona is located out of the reported range for presence of this species. Contact with the capital city of Cucuta is along a 75 km route and occurs mainly for commerce and commuting to work. Cucuta has an approximated population of 918,942. It is located on the border with Venezuela and represents one of the most endemic cities for DENV in the country [18]. Los Patios is a suburb of Cucuta and travel between Pamplona and Cucuta must necessarily pass through Los Patios.

### Human Sample Collection

#### Follow-up Study: Healthy Volunteers

In 2010, 49 non-native residents of Pamplona, with ages between 19 and 27 years old (χ = 22.3 years old), were enrolled in a follow-up study to test the concentration of anti-SGE antibodies before and after mosquito bite exposure. Pamplona is located at a higher altitude than the reported limit for *Ae. aegypti* in Colombia. Additionally, we performed mosquito collections in Pamplona during the study period in 2010 and no presence of *Ae. aegypti* was found. These individuals were selected because they traveled to Pamplona from DENV endemic areas in Colombia. Therefore, their exposure to the *Ae. aegypti* vector could be pinpointed to a particular travel date, given that serum was collected before leaving and upon their return to Pamplona. Travel occurred twice during the year for vacations. Serum was collected in 2010 before traveling for mid-year vacations (June [day 0]) and after returning to Pamplona (August [day 1], September [day 40] – November [day 80]).

#### Disease Risk: Febrile Patients

A total of 127 febrile individuals, with ages between 0 (6 to 11 months) to 80 years old (χ = 23.1 years old), with presumptive (clinical) DENV diagnosis between September and November of 2010 from Los Patios were included in a cohort to determine anti-SGE antibody concentration according to the dengue status from a single time point. The criteria to select patients from both hospitals were based on medical requests for DENV confirmation testing at the time of admission at the Local Hospital of Los Patios. DENV status and DENV serotype was determined by RT-PCR according to methods and using primers described elsewhere [19]. qRT-PCR conditions on the Roche LightCycler 480 were: RT Step: 48C for 5 min, 95C for 2 min. Amplification step: (95°C for 15sec, 60°C for 20sec) × 40 cycles and cool down: 4°C for 30sec. RNA from each DENV serotype was used as positive control (DENV1 strain WestPac-74 [Nauru Island 1974], DENV2 strain 1232 [Indonesia, 1978], DENV3 strain CH5548904500 [Thailand, 1973] and DENV4 strain LN 634441 [Malyasia, 1988]). Molecular grade water in the place of RNA was used as negative control during qRT-PCR runs. Presence of RNA viral (presence of dengue infection) is described as DENV positive (DENV (+)), and DENV negative (DENV (-)) if viral genome was not detected.

### Salivary Gland Extract Preparation

*Ae. aegypti* mosquitoes (Rockefeller strain) were reared at 25–28°C, 70–80% RH with a photoperiod of 16∶8 (L:D) h, and maintained on a 10% sucrose solution during adult stages. Female mosquitoes from 5 to 10 days old were cold-anesthetized, washed in 70% ethanol, and placed in PBS, pH 7.2, for salivary gland dissection. Salivary glands place in SGE buffer, a solution of PBS plus proteinase inhibitor (cOmplete ULTRA Tablets, Mini, EDTA-free, EASYpack, Roche Diagnostics, Indianapolis, IN) and were allowed to freeze at −80°C and thaw at 4°C four times to induce cell rupture and release of proteins; the resulting SGE was kept in PBS at −80°C until use [5], [6]. Protein concentration was determined using the Thermo Scientific NanoDrop™ (Thermo Fisher Scientific, Wilmington, DW) and the Bradford method (Bio-Rad protein assay).

### Anti-SGE antibody detection

Working conditions for the ELISA test were optimized according to our previous research [6]. Based on the results from the titration, 96-well ELISA plates (Nunc-Maxisorp, Nalgene Nunc International, Rochester, NY) were coated with 100 µL/well of 0.5 µg/ml of *Ae. aegypti* SGE prepared in coating solution (Kierkegaard and Perry Laboratories, Gaithersburg, MD) and incubated overnight at 4°C. Plates were blocked for 1.5 h with 5% dry milk in PBS (blocking buffer) (Invitrogen, Carlsbad, CA) at 37°C and incubated with 100 µL/well of 1/100 serum dilution in blocking buffer at 37°C for 2.5 h (filter paper blood lysate was incubated overnight at 4C). Plates were washed three times with wash solution (1× PBS and 0.1% Tween) and incubated with 100 µL/well of either goat anti-human IgG diluted 1∶1000 or IgM diluted 1∶10,000 horseradish peroxidase (HRP)-conjugated antibodies (Caltag Laboratories, Burlingame, CA) at 37°C for 1.5 h. Colorimetric development was obtained using 100 µL/well tetra-methyl-benzidine (TMB, one-solution microwell, Gene-Script, Piscataway, NJ) incubated for 15 min at room temperature. The reaction was stopped with 100 µL/well of stop solution (1 M phosphoric acid), and absorbance was measured at 450 nm. Each sample was tested in duplicate [4], [5]. Two controls were included in each plate: 1) control blank: two wells without SGE to control for nonspecific induction of color for any of the reagents used in the test; and 2) negative control: two wells with SGE but without human serum to control for any nonspecific color induction of the coating antigen.

### PAGE and Immunoblotting

Immunoblotting was performed with individual serum samples according to our previously published protocol [6] with minor modifications. During the optimization of our western blot protocol, we tested a DENV positive patient serum at 1∶100, 1∶300 and 1∶500 dilutions in blocking buffer (1× PBS with 1% Casein 0.1% Tween 20, Bio-Rad). We further tested dilutions of the IgG HRP conjugate at 1∶500, 1∶1000 and 1∶2000 in blocking buffer, optimized antibody and serum incubation temperatures at 4°C, 25°C, and 37°C, and optimized colorimetric development times of 3, 5, and 10 minutes. Optimal conditions for this assay were determined to be 1∶100 for the patient serum incubated for 2 h at 25°C, and 1∶1000 for the IgG HRP conjugate incubated for 1 h at 37°C. Briefly, 150 µg of SGE was mixed 1∶1 with 2× Laemmli buffer consisting of 65.8 mM Tris-HCl, pH 6.8, 2.1% SDS, 26.3% (w/v) glycerol, 0.01% bromophenol blue and 5% 2-mercaptoethanol. The SGE mixture was then loaded into the main well of a 12% preparative polyacrylamide minigel using Tris-Glycine-SDS (TGS) chemistry (Bio-Rad, Hercules, CA) along with 5 µL of a pre-stained molecular weight marker (Precision Plus Protein™ 10–250 kDa Kaleidoscope™, Bio-Rad) in the designated ladder well and electrophoresed at 90V for approximately 2.5 h and subsequently transferred using Trans-Blot® Turbo™ Transfer System to a polyvinylidene fluoride (PVDF) membrane (Mini PVDF Transfer Packs 7×8.5 cm PVDF membranes, Bio-Rad) utilizing the 7 min ‘Mixed Molecular Weight’ program. Membranes were blocked overnight with blocking buffer and incubated with individual human sera diluted 1∶100 in blocking buffer for 2 h at room temperature. Each membrane was washed 5 times with wash solution (1× PBS and 0.1% Tween 20, Bio-Rad) and incubated with HRP-conjugated Goat Anti-Human total IgG diluted 1∶1000 in blocking buffer for 1 h at 37°C. Color development was obtained with the HRP chromogenic substrate tetra-methyl-benzidine (TMB) (Novex®, Invitrogen). Band corrected density was measured using MyImageAnalysis Software version 1.1 (Thermo Fisher Scientific Inc., Rockford, IL). This software uses an algorithm to automatically select and identify lanes and band-boundaries for calculation of densitometry. As positive control, two or three DENV (+) individuals were included on each membrane.

### Data Analysis

Antibody concentrations were expressed as adjusted optical density (OD) calculated for each sample subtracting the mean OD value of the negative control and blank wells from the mean OD value of the duplicates for each sample. After verifying that our values did not meet the normal distribution (*p* = 0.0210; skewness and kurtosis normality test), the difference in the antibody concentrations between two independent groups (i. e. DENV (+) versus DENV (−) individuals) or between antibody concentrations in male versus females) was tested using the nonparametric Mann-Whitney *U* test. The difference between two dependent groups groups (i.e., day 1, day 40, and day 80) was assessed using the nonparametric Wilcoxon matched-pairs signed rank test. The Spearman's correlation coefficient was used to evaluate correlation of the concentration of IgM and IgG. All differences were considered significant with a probability of committing a type 1 error set at *P*<0.05. To measure risk by odd ratios (OR), the concentration of IgG anti-SGE saliva antibodies was transformed into categorical (high and low) using the median value (OD = 0.774) as breaking point). Age categories for the febrile patients were distributed in a way that have an approximately uniform number of individuals in each category based on the ages of the participants included in the study (0 to 80 years old (y.o.)). For instance, there are a total of 127 individuals distributed into 3 age categories (0–12 y.o. (n = 48), 13–30 y.o (n = 43) and >30 y. o. (n = 33)). Three individuals did not report their ages and were not included into the age vs. antibody concentrations analysis. For the protein analysis, we calculated the differences in the corrected density of protein band groups (represented by the average intensity per pixel for each defined band area minus a local background density correction) between DENV (+) and DENV (−) individuals. All statistical tests were computed using Prism version 5.02 (Graph Pad Software Inc., La Jolla, CA) and STATA™ version 10.1 (Stata Corporation, College Station, TX).

## Results

### Follow-up on Salivary Proteins Immunogenicity

**Figure 1:**
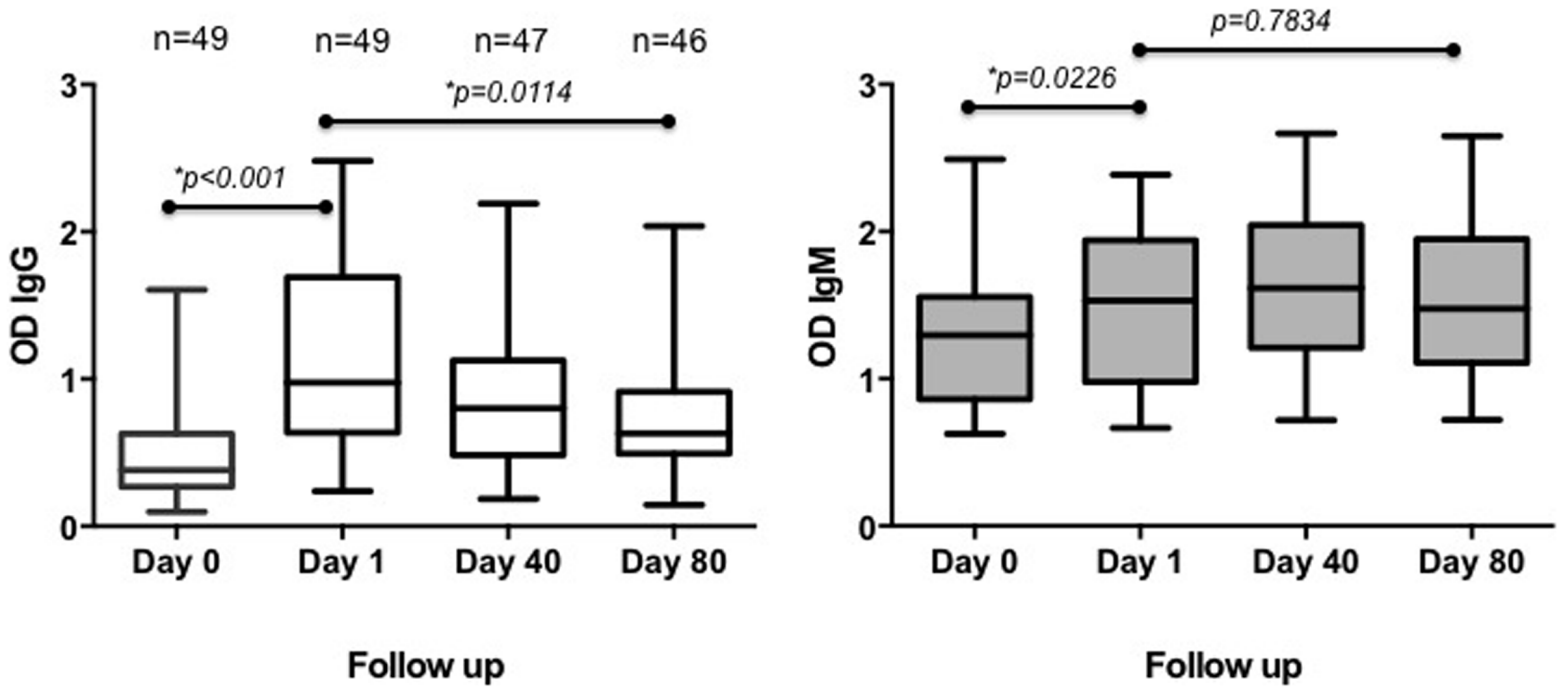
Follow-up study: Concentration of IgG and IgM antibodies of healthy individuals residing in Pamplona during the follow up. Representation in optical densities (OD) of the concentration of IgG antibodies against *Ae. aegypti* mosquito SGE. Statistical significance of Wilcoxon matched-pairs signed rank test represented as (*) p<0.05.

We designed a follow-up study (n = 49) to test the duration of the anti-*Ae. aegypti* SGE antibodies after exposure. In 2010, we collected sera from participant living in Pamplona where DENV is not endemic and where there is no known presence of *Ae. aegypti* mosquitoes. We sampled the individuals before and after traveling outside of Pamplona. Our results showed that the concentration of IgG and IgM anti-SGE antibodies were significantly lower before traveling than after returning from the vacation period (Wilcoxon matched-pairs signed rank test, p<0.0001 [IgG] and p = 0.0464 [IgM]). We observed a significant decrease in the concentration of IgG anti-SGE antibodies from day 1 to day 80 after returning to Pamplona (Wilcoxon matched-pairs signed rank test, p = 0.0053), while the concentration of IgM antibodies did not change significantly within the same period (Wilcoxon matched-pairs signed rank test, p = 0.9643) (**Figure 1**).

### Concentrations of IgG Anti-SGE Antibodies by DENV Fever Status

We collected a total of 127 serum samples from febrile individuals living in Los Patios. We found a relatively equal number of cases produced by DENV 1, 2 and 3 (34.8%, 34.8%, and 37%, respectively) and only one case attributable to DENV 4 (2.2%). (Proportions are greater than 100% due to the presence of mixed infections that were not taken into account in the subsequent analyses.)

**Figure 2:**
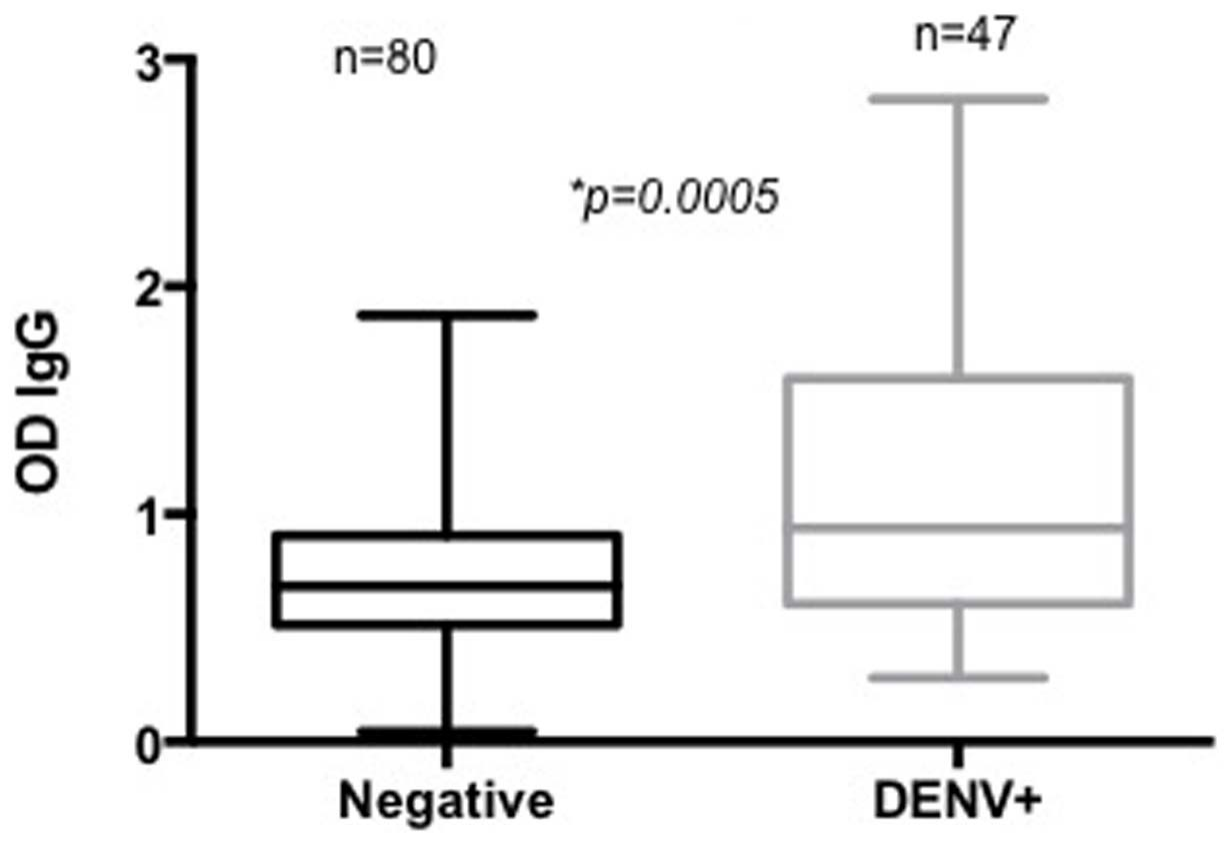
A: Concentration of anti-*Ae. aegypti* SGE antibodies according to the acute dengue infection status in Los Patios in subjects with DENV (+) (DENV+) and subjects without infection (DENV-). Statistical significance of Mann-Whitney test (*) p<0.05.

Regarding SGE-antibody concentrations, we found that these concentrations were significantly higher in DENV(+) individuals (n = 47) than in DENV(−) individuals (n = 80) (Mann-Whitney test, p = 0.0005) (**Figure 2**); Odd ratios showed that a person is 2.5 times more likely to be actively infected with DENV if the IgG concentrations of anti-*Ae. aegypti* SGE are high (95%CI: 1.1277–5.5409, p = 0.0289).

**Figure 3:**
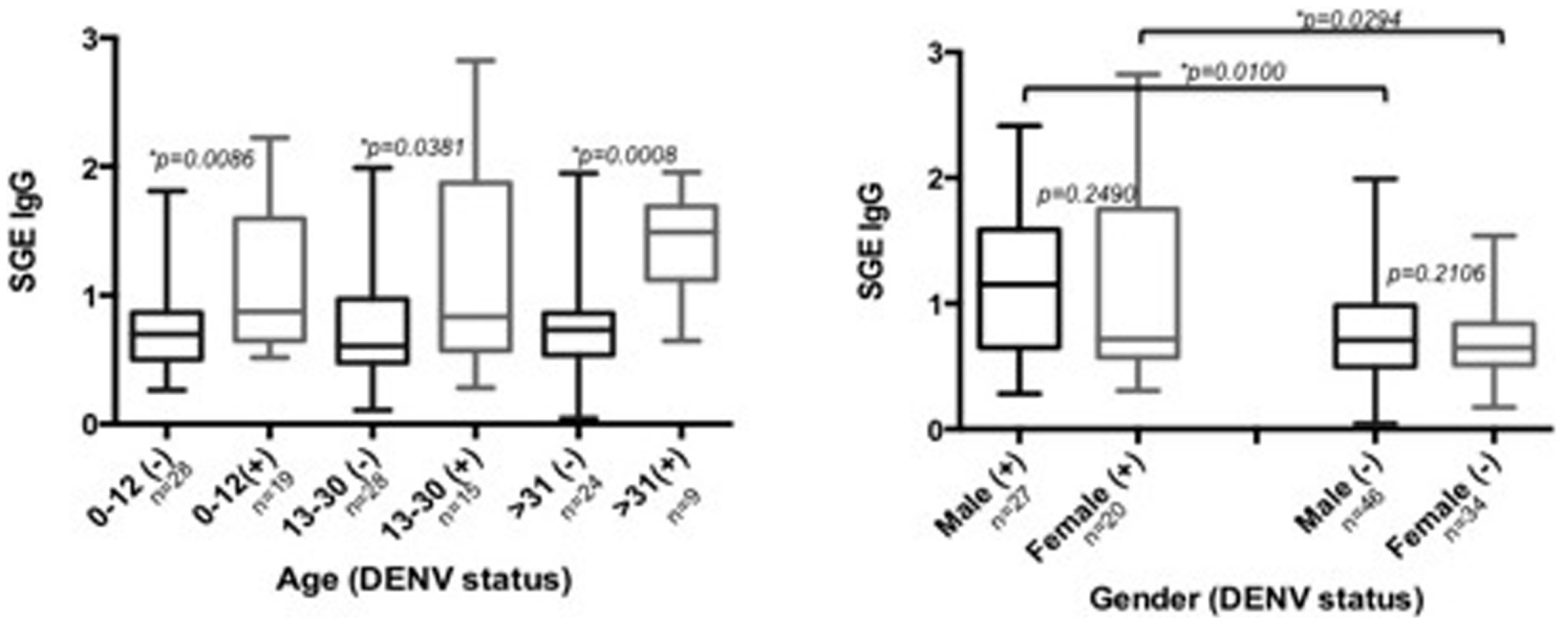
Concentration of anti-*Ae. aegypti* SGE antibodies according to gender and age categories in participants infected with DENV-RNA (+) versus participants without DENV-RNA (−) infection. Statistical significance of Mann-Whitney test (*) p<0.05.

**Figure 4:**
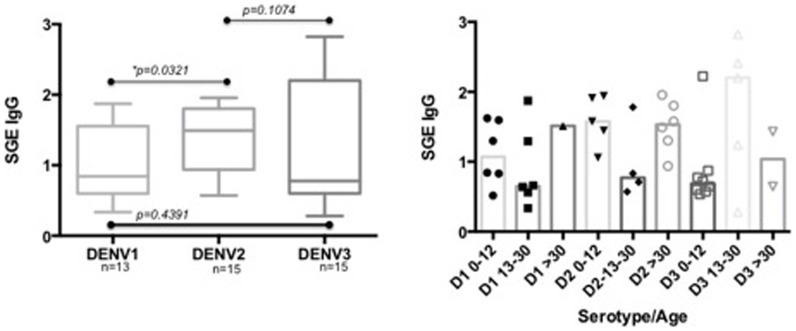
Concentration of anti-*Ae. aegypti* SGE antibodies according to the DENV serotype in participants from Los Patios diagnosed by RT-PCR. Figure also represents the distribution of the different SGE-antibody concentrations for each DENV serotype by age group. Statistical significance (*) p<0.05.

In order to determine if these differences were influenced by age or gender, we calculated the differences between male and females within the DENV (+) or DENV (−) individuals and found no difference (Mann-Whitney test, p = 0.2490 and p = 0.2106, respectively). Interestingly, these differences were significant when we compared females DENV (+) against female DENV (−) (Mann-Whitney test, p = 0.0294) and when we compared males DENV (+) against male DENV (−) (Mann-Whitney test, p = 0.0100) (**Figure 3**). Similar results were obtained when comparing each age category within the DENV (+) and the DENV (−) groups. Again, antibody concentrations were significantly higher in the DENV (+) patients. We sorted the DENV positive individuals into each one of the serotypes circulating in the area; we did not find significant differences in the IgG anti-SGE antibody concentrations among the groups (Mann-Whitney test, DENV2 vs. DENV3 p = 0.1074 and DENV1 vs. DENV3 p = 0.4391) with the exception of DENV2 vs. DENV1 (Mann-Whitney test, p = 0.0321) (**Figure 4**). We did not perform this analysis on patients with DENV4 (n = 1) and mixed infections (n = 3) due to their very low sample size.

### Protein Detection by Immunoblotting

**Figure 5:**
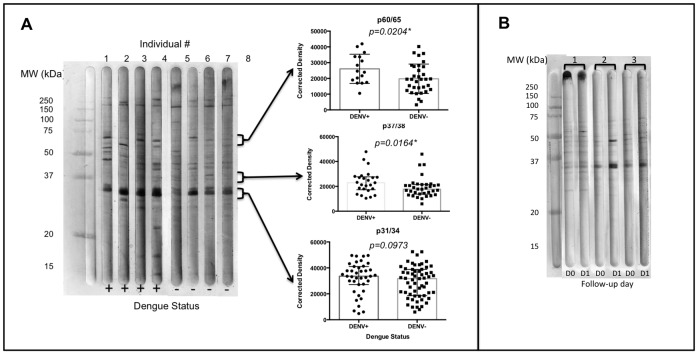
Representative western blot results from four DENV-RNA (+) and four DENV-RNA (−) individuals from Los Patios, with corresponding statistical analysis of 104 (DENV-RNA (+) (n = 41), DENV-RNA (−) (n = 63)) samples tested. Statistical significance (*) p<0.05 (A). Western blot results from three non-febrile healthy individuals from Pamplona on day 0 (before travel to endemic area (DO)) and day 1 (after travel (D1)). Numbers represent each subject (B).

A sub-sample of 106 individuals from Los Patios and 9 samples from Pamplona were selected for the testing of specific immunogenic proteins. *Ae. aegypti* SGE proteins with molecular weights from 15 to >250 kDa were recognized by the study subjects. We observed high reactivity to at least eight groups of proteins: p60/65, p48/47, p37/38, p35/36, p31/34, p28/30, p20/24 and p17/19. Proteins with in the molecular weight group of 31/34 kDa were recognized by the majority of individuals and we did not observed any significant difference in the corrected density between DENV (+) and DENV (−) subjects (Mann-Whitney test, p = 0.0973) (**Figure 5a**). However, band corrected density was significantly higher in DENV (+) subjects for proteins with molecular weight of 60/65, 37/38 and 20/24 (Mann-Whitney test, p<0.05). Participants with immunoreactivity to p35/36 were 2.9 times more likely to have active DENV infection (95%CI: 1.024–8.3662, p = 0.0198).

In addition, we selected nine non-febrile individuals from Pamplona to identify specific immunogenic proteins in this group on day 0 and day 1. We found significantly higher protein corrected density values on day 1 (after travel) compared to day 0 (**Figure 5b**) (Mann-Whitney test, p<0.05).

## Discussion

In Colombia, dengue fever and its severe forms are major public health threats [20]. The Pan-American Highway is one of the main routes for commerce in Colombia. This highway runs through the state of Norte de Santander and our study area. Given that transport and commerce are principal factors in the spread of not only mosquitoes but the diseases they carry [21], it follows that multiple DENV outbreaks have been observed in this state in the last decade [22]. In 1997, Paluso et al. found that seasonal exposure to mosquito bites induces an increase in anti-mosquito SGE antibodies [23]. Other studies have also shown that anti-mosquito SGE antibody concentrations are short-lived and indicative of recent mosquito contact [24]–[26]. Our results further confirm the waning of anti-mosquito SGE IgG when exposure to that mosquito species is not sustained. The concentration of IgG antibodies against *Ae. aegypti* SGE decreased over the follow up months while the concentration of IgM antibodies remained mostly unchanged.

*Ae. aegypti* mosquitoes have not been reported in Colombia on altitudes above 2,200 m.a.s.l. However, mosquito surveillance performed by our research team in 2010 did find non-*Ae. aegypti* species. Several studies have shown that IgG antibodies have a greater specificity and can recognize species-specific SGE antigens with low cross reactivity among vectors [5], [6]. Indeed, previous data have showed minimal antigen cross reactivity between several mosquitoes including *Ae. aegypti* and *Culex quinquefasciatus* [16]. Additionally, the decreasing trend in IgG antibody concentrations showed buy subject living in Pamplona under the absence of *Ae. aegypti* bites supports the evidence of low cross-reactivity between the SGE antigen from the former two mosquito species. While specificity can also be a characteristic of IgM anti-SGE antibodies, other studies have shown the possibility of cross-reactivity, especially when using whole SGE for the ELISA-based test antigen [27]–[29]. Thus, the sustained IgM concentrations against *Ae. aegypti* SGE is likely due to the less specific nature of IgM antibodies and could be the result of the exposure to mosquito bites of other culicine mosquitoes such as *Culex quinquefasciatus*, which were found in Pamplona.

Our study showed that the concentration of anti-SGE IgG antibodies was significantly higher in viremic participants with confirmed DENV infections by RT-PCR versus uninfected participants. Similar associations have been observed in other vector-borne diseases, including malaria and leishmaniasis [6], [30]–[33] demonstrating that anti-vector saliva antibodies are a reliable biomarker for vector activity and disease transmission.

IgG anti-SGE concentrations in participants with DENV2 infections were significantly different from the concentrations of anti-SGE antibodies of participants infected with the DENV 1 and 3. We found a similar number of individuals infected by each these serotypes: DENV1 (n = 13), DENV2 (n = 15) and DENV3 (n = 15), therefore our differences are likely not explained by differences in sample size but by the frequency of mosquito exposure for DENV2 infected participants. The majority of participants with DENV2 in our study sample (9/15) were older than 31 y.o., the age comparison results for IgG anti-SGE concentrations showed that this particular age category presented, not significant, but still somewhat higher IgG concentrations than the other two age categories. Alternate hypothesis could include questions of relative fitness of DENV 2 relative to the other two serotypes, and is subject of current investigations in our laboratory.

Immune response to vector saliva is complex and can differ from one individual to another [26]. We tested individual serum samples to have a better picture of the proteins recognized by our study group. We observed the highest immunoreactivity in proteins with molecular weights between ∼70 and 15 kDa. Other work has reported the immunogenicity of *Ae. aegypti* salivary proteins with similar molecular weights [26], [30]. Elanga et al. described a ∼34 kDa protein as a putative marker for *Ae. aegypti* bites. They have found that the concentration of IgG antibody response to Nterm-34 kDa salivary peptide may reflect the real intensity of human exposure to *Ae. aegypti* bites [34]. Our study not only shows the reactivity to a 34 kDa but to a group on protein in the range form 31 to 34 kDa.

A 37 kDa protein has been reported as one of the most abundant on *Ae. aegypti* salivary protein as well as one recognized in a very high proportion for patients with dengue fever [30] It is believed that this protein belongs to the D7 protein family involved in inflammation and platelet aggregation [26] it is also believed that inhibition of this protein does not confer protective immunity against DENV disease [30] but it could be useful marker of recent exposure to *Ae. aegypti*. We found a significantly higher immune response to proteins with similar molecular weight in our DENV positive subjects. Likewise, proteins of 35/40 and 61/67 kDa has been documented to be expressed more by dengue infected mosquitoes [35]. We found greater immune response to p60/65 and p37/38 in DENV positive individuals. It is possible that our p60/65 includes, among others, the apyrase which is reported to have molecular weight of ∼68 kDa [26]. It is believed that antibodies against apyrase may increase dengue transmission risk by increasing mosquito probing time and number of bites in order to obtain the blood meal [13], [14].

In conclusion, the concentrations of anti-*Ae. aegypti* SGE antibodies can be used as a tool to measure risk for DENV in regions with intense transmission. Logically, the risk of disease transmission increases with increased human-vector contact and, given the waning nature of anti-SGE IgG, increased concentrations of IgG require boosting and necessarily indicate recent contact.
